# Feasibility of evidence-based diagnosis and management of heart failure in older people in care: a pilot randomised controlled trial

**DOI:** 10.1186/1471-2318-12-70

**Published:** 2012-11-14

**Authors:** Helen C Hancock, Helen Close, James M Mason, Jeremy J Murphy, Ahmet Fuat, Mark de Belder, Trudy Hunt, Andy Baker, Douglas Wilson, A Pali S Hungin

**Affiliations:** 1 Durham Clinical Trials Unit, School of Medicine, Pharmacy and Health, Durham University, Queen’s Campus, Wolfson Research Institute, University Boulevard, Stockton-on-Tees, TS17 6BH, United Kingdom; 2 School of Medicine and Health, Durham University, Queen’s Campus, Wolfson Research Institute, University Boulevard, Stockton-on-Tees, TS17 6BH, United Kingdom; 3 Department of Cardiology, Darlington Memorial Hospital, County Durham and Darlington NHS Foundation Trust, Hollyhurst Road, Darlington, DL3 6HX, United Kingdom; 4 Department of Cardiology, The James Cook University Hospital, South Tees Acute Hospitals NHS Foundation Trust, Marton Road, Middlesbrough, TS4 3BW, United Kingdom; 5 Department of Cardiology, North Tees Hospital, North Tees and Hartlepool NHS Foundation Trust, Hardwick Road, Stockton, TS19 8PE, United Kingdom

**Keywords:** Chronic heart failure, Treatment outcomes, Randomised controlled trial, Older people, Long-term care facilities

## Abstract

**Background:**

Many older people in long-term care do not receive evidence-based diagnosis or management for heart failure; it is not known whether this can be achieved for this population. We initiated an onsite heart failure service, compared with ‘usual care’ with the aim of establishing the feasibility of accurate diagnosis and appropriate management.

**Methods:**

A pilot randomised controlled trial which randomised residents from 33 care facilities in North-East England with left ventricular systolic dysfunction (LVSD) to usual care or an onsite heart failure service. The primary outcome was the optimum prescription of angiotensin-converting enzyme inhibitors and beta-adrenergic antagonists at 6 months.

**Results:**

Of 399 echocardiographically-screened residents aged 65–100 years, 30 subjects with LVSD were eligible; 28 (93%) consented and were randomised (HF service: 16; routine care: 12). Groups were similar at baseline; six month follow-up was completed for 25 patients (89%); 3 (11%) patients died. Results for the primary outcome were not statistically significant but there was a consistent pattern of increased drug use and titration to optimum dose in the intervention group (21% compared to 0% receiving routine care, p=0.250). Hospitalisation rates, quality of life and mortality at 6 months were similar between groups.

**Conclusions:**

This study demonstrated the feasibility of an on-site heart failure service for older long-term care populations. Optimisation of medication appeared possible without adversely affecting quality of life; this questions clinicians’ concerns about adverse effects in this group. This has international implications for managing such patients. These methods should be replicated in a large-scale study to quantify the scale of benefit.

**Trial registration:**

ISRCTN19781227 http://www.controlled-trials.com/ISRCTN19781227

## Background

Evidence-based management of heart failure (HF) reduces mortality and morbidity and improves quality of life. The benefits of drug management for HF have been extensively researched and are included internationally in guidance for the management of HF in older people, though these do not specifically refer to those in long-term care [1-6]. Both angiotensin-converting enzyme inhibitors (ACEi) and beta-adrenergic antagonists (β-blockers) reduce all cause mortality by 20-25%, delay disease progression, and reduce symptoms and signs of HF [7-9]. However, many patients in long-term care may not be managed in line with evidence based guidelines [10-15]. The reasons for this remain unclear but may be due in part to the increased requirements for monitoring, burden of comorbidity, cognitive deficit, and polypharmacy in the elderly [4]. Despite these challenges, evidence based management appears to be as effective in this group as in the general population [2,16]. The use of ACEi and β-blockers to treat HF in older people living in their own homes or in long term care are associated with reduced hospitalisation and mortality rates [16-19]. The scale of benefit for ACEi was between 10% [18] and 33% [19] reduction in risk of death and for β-blockers was a 5% reduction in all cause mortality [20] and a 27% reduction in combined risk of death or hospitalisation [16]. Despite these benefits, there appears to be a tendency to under-prescribe in long-term care [21-23]. The decline in research in the last decade suggests that appropriate therapeutic management of HF in the long-term care population has fallen from the research agenda.

Variations in HF management in the long-term care population may be due in part to the difficulty accessing specialist care [24]. Difficulties in differential diagnoses, knowledge about the benefits of ACEi compared to diuretics, and the inconvenience of monitoring and adverse effects are identified as key challenges [25,26]. Personal preferences [21,27] and ageist values are also recognized by general practitioners (GPs) as contributing to variations in practice [26]. Although research indicates the challenges of HF management in primary care, little is known about the most appropriate organisation of care to improve care delivery for residents in care homes. This pilot trial evaluates the implementation of a HF team delivering onsite assessment and management, comparing outcomes with routine GP care. A nested qualitative element (This paper is under consideration by BMC Geriatrics) evaluated patients’ and clinicians’ experiences of the model. Findings suggest this as an acceptable solution to variations in the management of heart failure for this group.

## Methods

### Trial design

A pilot randomised controlled trial using a PROBE design (prospective, randomised, open-label, blinded end point), compared two models of care: routine GP-led care or an onsite HF team.

### Participants

Residents from 33 of 35 long-term care facilities (UK residential and nursing homes) in Teesside, North East England, aged ≥65 years without terminal disease and who were permanently resident were eligible to participate (see [28] for full details). In the UK, residents are assessed for their suitability for one of three types of long-term care: residential homes provide personal and social care for people no longer able to live independently; nursing homes provide medical and nursing care in addition to these services and some also provide specialist mental health care. No exclusions were made on the basis of cognitive capacity, comorbidities or immobility. Initial and process consent was sought directly from the resident, or from their relative (or a consultee) when a resident lacked the capacity to provide informed consent. Capacity to consent was determined by the mini mental state examination (MMSE) [29] and process consent using the abbreviated mental tests score (AMTS) [30] prior to assessments. 529 residents (of 1701) were judged by care facility managers to be ineligible predominantly due to concerns over the balance of risks and benefits of participating. Of the remaining 1172 eligible residents, consent for participation in the prevalence study was obtained for 405 (35%); this formed the basis for recruitment to this pilot trial. The primary reason for non-participation was relatives declining on behalf of residents due to similar concerns.

We extracted anonymised demographic details (date of birth, gender and ethnicity) of all eligible residents (including non-participants) in order to assess the representativeness of participants and thus the potential for selection bias. Study data were collected over a 14 month period from April 2009.

### Assessment

Participants underwent a diagnostic assessment within each facility, including MMSE [28], demographic information, medication, and past medical history, quality of life assessment (EuroQol: EQ-5D and EQ-VAS) [31], physical examination, electrocardiography, echocardiography, [32] and blood sampling. Blood tests included standard U&Es, FBC, LFTs, Glucose, TSH, hsCRP, Troponin I, natriuretic peptides, and novel biomarkers: mid-regional pro atrial natriuretic peptide (MR-proANP), mid-regional pro adrenomedullin (MR-proADM) and C-terminal provasopressin (Copeptin) (test performance is reported in a separate diagnostic accuracy study paper [33]). All assessments were conducted by the study team; echocardiography, ECG and physical assessment findings were blinded from each other; findings were reviewed by a consultant cardiologist (JJM) who made the definitive diagnosis of heart failure (LVSD or HFpEF). Findings were subsequently reviewed by a second heart failure specialist (AF) who was blinded to the diagnosis (with 100% agreement). On completion of the study 12.5% (50) of echocardiograms were randomly selected and independently reported by a BSE-accredited cardiac physiologist (not involved in the study) to test reliability and validity of findings (with 100% agreement).

Participants identified using echocardiography as having HF due to left ventricular systolic dysfunction (LVSD) were screened for trial eligibility. Any resident not already prescribed both an ACEi and β-blocker at optimal dose for LVSD was eligible. Residents with an existing diagnosis of LVSD were eligible; those with heart failure with preserved ejection fraction (HFpEF) or with terminal disease were ineligible. Written consent was sought from residents directly or through a consultee when residents lacked cognitive capacity.

### Interventions

Residents randomised to the HF service received an assessment visit by a consultant cardiologist who initiated a plan of treatment, followed by visits at one to two weekly intervals within the home by heart failure specialist nurses (HFSNs). The HFSNs enacted the plan, including blood tests, assessment of symptoms and signs, educational advice, and medication titration. The team cardiologist was contacted in the event of any change in a resident’s health status related to heart failure. GPs were notified of other anomalies on the same day as they were detected. Residents were treated according to NICE guidance [3] using drugs licensed for HF. Ramipril (ACEi) and bisoprolol (β-blocker) were used as standard since these are simple to titrate and have limited cost implications. Spironolactone was included for patients with New York Heart Association (NYHA) [34] class IV, or NYHA class III if the participant remained symptomatic on other treatments. Each drug was optimised according to guidance unless clinically contraindicated or declined by the patient or their consultee.

The team notified primary care providers of any alterations to HF management. Drug prescriptions were managed using usual practice within the care home and the associated general practice. In addition, each patient’s GP was notified by letter each time they were reviewed by the HF team, detailing symptoms and signs as well as medication changes. Once HF management was optimised, participants were discharged from the HF service and a final letter was sent from the team consultant cardiologist, in consultation with a GP with a specialist interest in HF, to the relevant GP notifying current management and advice about ongoing care. For those residents randomised to routine care, echocardiographic test results were communicated to the resident and a letter sent to their GP from the team consultant cardiologist outlining a personalised HF management plan.

### Sample size

The original (full trial) sample size was based on a difference in proportions receiving ACEi and β-blockers (40% and 70%) and required a total enrolment of 100 residents with LVSD (Fisher's exact test, α=0.05, power=80%). Allowing for non-participation or loss to follow-up, the study aimed to identify 125 eligible residents. Estimates from previous literature suggested a prevalence of heart failure of 25%, thus the prevalence study required 500 residents in order to identify sufficient eligible residents for the trial. However, previous literature did not differentiate between LVSD and HFpEF – using agreed criteria, screening identified an 8.5% prevalence of LVSD heart failure (and 14.3% HFPEF), rendering the original sample size unfeasible. In response the trial was reconstituted as a pilot study retaining the original trial design and per protocol analyses but aiming to recruit 25–30 participants. Although underpowered to address the primary endpoint, this level of recruitment would show a difference of optimally treated patients (prescribed both ACEi and β-blockers) of 50% (assuming a change from 25% to 75%, chi-square test).

### Randomisation

Consenting residents were randomly assigned to either usual care or the HF service. Randomisation used stratified blocks according to NYHA classification [34] (where: Mild HF = NYHA class I, Moderate HF = NYHA classes II & III, Severe HF = NYHA class IV) and by care home. Treatment allocation was concealed until formal process consent was obtained from the resident (or their consultee).

### Outcomes

Participating homes and general practices provided access to patient records to establish changes in prescribing, and HF related events (hospitalisation and mortality). All participants’ notes were reviewed at 6 and 12 months by a blinded assessor. Residents in the intervention arm were discharged back to usual care by 6 months, or at the point of optimal titration if before 6 months. The primary trial outcome was the proportion of residents receiving the guideline-recommended dose of prescribed ACEi and β-blockers at 6 months after enrolment. Titration to a percentage of the recommended (theoretical) optimum provided a consistent and measurable benchmark for comparing groups, where it was not assumed that 100% was achievable or necessarily desirable for the individual resident. Recognising that the optimal dose would vary for individual patients according to response and tolerability, we conducted secondary analysis on residents receiving ‘any dose’.

The optimum dose was defined as ramipril 10mg and bisoprolol 10mg or equivalent; use of an angiotensin receptor blocker (ARB) was counted as equivalent to an ACEi. Since the prescription of spironolactone involved some judgement on the part of the treating physician this was not included in the primary outcome but rates were recorded for both groups, with a target dose of 50mg spironolactone. Secondary analyses included: use of individual drug classes at any dose; the proportion of patients dying or being hospitalized for HF; acceptability to residents of the two forms of care; changes in functional capacity and quality of life (using EQ-5D and EQ-VAS [31])*.*

### Analysis

Data analysis was conducted by intention to treat and using SPSS version 19. Differences in proportions were analysed using Fisher’s exact test, cross tabulations of more than two categories using Pearson chi-square, and continuous variables using unpaired Student’s t-test.

### Ethical approval

This study was approved by Durham University and Leeds (West) national research ethics committees. The investigation conforms with the principles outlined in the Declaration of Helsinki.

## Results

### Baseline characteristics

Of 399 residents screened in the HFinCH prevalence study, 34 (8.5%) were diagnosed with LVSD (19 (56%) mild, 9 (27%) moderate, and 6 (18%) severe). Of these, four were ineligible (three were not symptomatic, and one had a terminal condition), thus 30 were eligible for the trial and 28 (93%) agreed to participate. Of the two patients who declined: one was due to relocate and one did not wish to participate.

The mean age of trial subjects was 83.6 (range 70–98), all were white British and 12 (43%) were male. Resident data was collected from 24 GPs based at 11 different medical centres. Residents were predominantly categorised as requiring residential care (17; 61%) rather than nursing care; 17 care homes were involved in the trial. Twelve residents (43%) were unable to give consent due to cognitive impairment and a consultee declaration was obtained.

Participants were randomised to either the HF service (intervention group, N=16), or to routine care (control group, N=12): the two groups were similar statistically at baseline in demographic characteristics, HF severity, comorbidities, signs and symptoms, and prescribed drug use (Table 1). Individual GPs did not have patients in both arms of the trial. At baseline, 46% of patients were receiving an ACEi (of these 77% were prescribed ramipril, 15% lisinopril, and 8% perindopril), 50% were receiving a β-blocker (of these 42% were prescribed bisopropol, 36% metoprolol and 22% atenolol); 32% were receiving both, although none at the target dose; no patients were prescribed spironolactone.

**Table 1:** Baseline characteristics of participants

		**Control N**_**A**_**= 12**	**Intervention N**_**B**_**= 16**	**P value (A-B)**
**Demographics**				
Age (y)^1^		81.8 (7.1, 71–94)	85.1 (6.7, 70–98)	0.233
Gender	Male:Female	5:7	7:9	0.609
Ethnicity	White British	12 (100%)	16 (100%)	-
Care Home Type^2^	N:R:D	5:7:0	3:10:3	0.171
Body Mass Index^1^		25.5 (4.6, 20.8-36.4)	27.7 (4.8, 19.5-35.9)	
**Heart failure**				
Heart Failure^3^	Confirmed:New	6:6	9:7	1.000
NYHA^4^ class	I:II:III:IV	5:4:1:1	10:1:4:1	0.213
Ejection Fraction (mean %, SD)		43 (6.3)	33 (1.4)	0.146
**Total no of co-morbidities (mean, SD)**^5^		4.67 (2.1)	4.50 (1.5)	0.319
**Renal Function**				
Urea Abnormal^6^		8 (75%)	9 (56%)	0.705
Creatinine Abnormal^7^		2 (17%)	5 (31%)	0.558
**Prescribed drugs**^8^				
ACEi and β blocker		3 (25%)	6 (38%)	0.687
ACEi		6 (50%)	7 (44%)	1.000
β blocker		5 (42%)	9 (56%)	0.704
Angiotensin Receptor Blocker		0 (0%)	0 (0%)	-
Calcium Channel Blocker		1 (8%)	4 (25%)	0.355
Diuretic		8 (67%)	9 (56%)	0.705
Statin		7 (58%)	8 (50%)	0.718
Digoxin		4 (33%)	1 (6%)	0.133
Antiplatelet		7 (58%)	10 (63%)	1.000
Spironolactone		0 (0%)	0 (0%)	-
Bronchodilators		4 (33%)	2 (13%)	0.354
Warfarin		3 (25%)	1 (6%)	0.285
Non Steroidal Anti-Inflammatory Drugs		0 (0%)	1 (6%)	1.000
Total no of prescribed drugs (mean, SD)		10.3 (3.4)	9.5 (4.7)	0.287

1 Mean (standard deviation, range).

2 Nursing:Residential:Dementia.

3 Confirmed cases pre-existed in general practice HF register records.

4 New York Heart Association [34].

5 From a predefined list of co-morbidities (MI, IHD, Hypertension, AF, valvular heart disease, diabetes, COPD, osteoarthritis, cognitive impairment).

6 Outside normal limits (2.5-7.0mmol/l).

7 Outside normal limits (50-110μmol/l).

8 As recorded in GP notes.

### Follow-up

**Figure 1:**
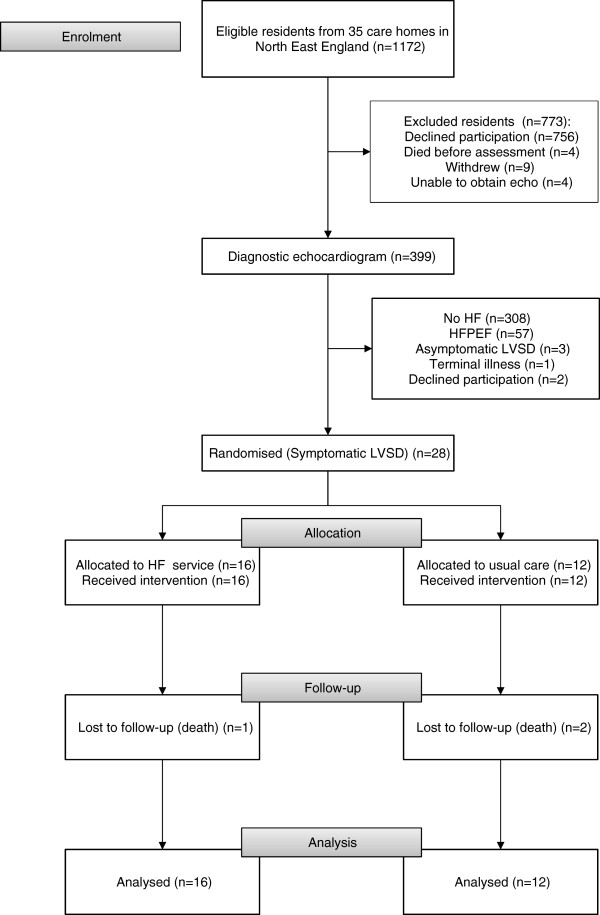
**Participant Flow Diagram.** Flow diagram showing enrolment, recruitment, allocation, follow-up and analysis numbers for the trial in accordance with the CONSORT statement [30].

Six month follow-up was completed by 25 residents (86%); the remaining 3 (14%) died during follow-up (2 from the intervention group, 1 from usual care). See Figure 1 for a (CONSORT [35]) flow diagram showing enrolment, recruitment, allocation, follow-up and analysis numbers for the trial. All but four participants in the intervention group were assessed within 4 weeks of HF diagnosis by a consultant cardiologist and a plan developed in collaboration with a HF specialist nurse; the remaining four were seen within 8 weeks (due to consultees considering process consent). The cardiologist approved a full titration plan at the first visit for 13/16 patients (81%); of the remainder, 1 patient was unsuitable for ACEi titration and 1 for β-blocker titration due to previous adverse reactions, and 1 had delayed commencement on ACEi to allow a previous adverse reaction to be ruled out. The exact point of titration for the usual care group was not routinely specified in general practice records.

At six months, 3 (21%) of the intervention group achieved optimal doses of combined ACEi and β-blockers compared to 0 (0%) in the control group (p=0.250, see Table 2). Of intervention patients, 7 (50%) reached a lower maximum dose tolerated, although no comparable data is available for the control group. Reasons for failure to reach full titration for ACEi were deterioration in renal function (n=3), hypotension (n=1), patient choice (n=1); and for β-blockers reasons were bradycardia (n=6), dizziness and confusion (n=1), hypotension (n=1), patient choice (n=1), tiredness (n=1). On one occasion, a β-blocker was discontinued during a hospital admission; this was recommenced following discussion between the HF service and the GP. Use of ACEi and β-blocker at any dose was achieved in 10 (71%) of patients in the intervention group and for 5 (45%) of residents in usual care. All residents in the intervention group received at least one step change increase in dose or prescription of both ACEi and β-blocker. Of all residents in usual care, 3 (27%) received no increase in ACEi use and 8 (72%) no increase in β-blocker use; a further 2 (18%) residents had their ACEi dose reduced at 6 months.

**Table 2:** Drug treatment and morbidity at 6 and 12 months

	**Control n**_**A**_**=11**	**Intervention n**_**B**_**= 14**	**P value (A-B)**
**6 months Use at optimum dose**			
ACEi + β blocker	0 (0%)	3 (21%)	0.250
ACEi	3 (27%)	8 (57%)	0.414
Ramipril	2 (18%)	6 (43%)	0.402
β blocker	2 (18%)	3 (21%)	1.000
Bisoprolol	0 (0%)	3 (21%)	0.250
Spironolactone	0 (0%)	0 (0%)	-
**Use at any dose**			
ACEi + β blocker	5 (45%)	10 (71%)	0.442
ACEi	5 (45%)	13 (93%)	0.075
Ramipril	4 (36%)	11 (79%)	0.122
β blocker	7 (64%)	12 (86%)	0.653
Bisoprolol	5 (45%)	11 (79%)	0.397
Spironolactone	0 (0%)	2 (14%)	0.500
**Morbidity**			
Hospitalisations at 6 months for HF	0 (0%)	0 (0%)	-
Hospitalisations at 6 months for CVD	0 (0%)	0 (0%)	-
Hospitalisations at 6 months for any cause	2 (18%)	1 (7%)	0.498
**12 months (post-trial)**	**Control n**_**A**_**= 8**	**Intervention n**_**B**_**= 13**	**P value (A-B)**
**Use at optimum dose**			
ACEi + β blocker	0 (0%)	1 (8%)	1.000
ACEi	3 (38%)	5 (38%)	1.000
Ramipril	3 (38%)	4 (31%)	1.000
β blocker	0 (0%)	1 (8%)	1.000
Bisoprolol	0 (0%)	1 (8%)	1.000
Spironolactone	0 (0%)	0 (0%)	-
**Use at any dose**			
ACEi + β blocker	5 (63%)	7 (54%)	1.000
ACEi	6 (75%)	11 (85%)	0.609
Ramipril	5 (63%)	10 (77%)	0.376
β blocker	5 (63%)	7 (54%)	1.000
Bisoprolol	5 (63%)	7 (54%)	1.000
Spironolactone	0 (0%)	2 (15%)	0.494
**Morbidity**			
Hospitalisations at 12months for HF	0 (0%)	0 (0%)	-
Hospitalisations at 12 months for CVD	0 (0%)	0 (0%)	-
Hospitalisations at 12 months for any cause	2 (25%)	3 (23%)	1.000

Each patient was assessed for the proportion of optimal treatment received, as a combination of ACEi and β-blockers. The proportion of optimal treatment received was 51.6% (SD 30.6%) for the intervention group and 28.7% (SD 29.4%) for the usual care group (p=0.057).

Taking each drug in turn, more intervention patients received an ACEi at optimum dose (57% vs 27%, p=0.414), while numbers receiving a β-blocker at optimum dose were similar (21% vs 18%, p=1.000). No participants in either group received the optimum dose of spironolactone but two patients (one with moderate LVSD, one with severe) in the intervention group received spironolactone at a lower dose. Comparing the intervention and usual care groups at six months, there were non-statistically significant increases in ACEi use at any dose (93% vs. 45%, p=0.075) and β-blocker use at any dose (86% vs. 64%, p=0.653).

The mean time from initial specialist assessment to optimum titration in the intervention group was 4 months (range 1– 6 months) with a mean of 5 titration steps (range 1–15) to optimum or maximum tolerated dose. It was not possible to assess the mean time in usual care from available records.

Following the end of the trial, general practice records were assessed at 12 months to establish treatment fidelity and changes in management. Twelve month follow-up was completed by 21 residents (75%); 6 (21%) died during follow-up (3 from the intervention group, 3 from usual care, p=1.000) and 1 (4%) was lost to follow-up. At 12 months there were no significant differences between HF treatment for each group, and no differences in hospitalisation rates. Comparing 6 to 12 month prescribing trends, there was a substantial reduction in both numbers and doses of β-blockers and ACEi among those discharged from the intervention group (for example, patients receiving a prescription of a β-blocker at any dose fell by 32%, and ACEi at any dose fell by 8%). Prescribing trends in the usual care group remained largely stable although at 12 months all β blocker prescriptions were for bisoprolol and 90% of ACEi prescriptions were for ramipril, with the remainder being for lisinopril and perindopril. In the intervention group 3 of 16 patients (21%) were titrated to optimal dose by the HFSN before being discharged from the HF service but this had fallen to 1 of 16 (6%) by 12 months.

### Morbidity

There was no significant patient morbidity (proxied using hospitalisation during follow-up (Table 2)). At 6 month follow-up, 2 (18%) participants in the usual care group and 1 (7%) in the intervention group had a hospital admission (for cholecystitis, epistaxis, and ophthalmic surgery respectively). MMSE, EQ-5D and EQ-VAS values, as measures of cognitive impairment and health-related wellbeing were similar at baseline and 6 months, comparing groups; for each, higher scores indicate better health or cognitive status (Table 3).

**Table 3:** Cognitive impairment and quality of life

	**Baseline (mean, standard deviation, range)**			**6 months (mean, standard deviation, range)**		
	**Control N**_**A**_**= 12**	**Intervention N**_**B**_**= 16**	**P value (A-B)**	**Control N**_**A**_**= 11**	**Intervention N**_**B**_**= 14**	**P value (A-B)**
**MMSE**^**1**^	20.3 (10.4, 0–30)	18.9 (9.3, 2–30)	0.297	18 (11, 0–29)	20 (10, 1–30)	0.512
**EQ-5D**^**2**^	0.66 (0.27, 0.09-1)	0.58 (0.25, 0.08-1)	0.270	0.59 (0.35, -0.016-1)	0.58 (0.30, 0.0-1)	0.574
**EQ-VAS**^**2**^	63 (18.3, 50–100)	73 (18.0, 50–100)	0.421	66 (16, 40–80)	62 (23, 10–80)	0.640

1 Mini Mental State Examination [29]: The MMSE comprises 11 main questions with a maximum score of 30 points. Higher scores indicate better cognitive status. A score of 23 or less indicates a lack of capacity to provide informed consent for research purposes.

2 Components of EuroQol © [31]: The EQ-5D comprises five questions on mobility, self care, pain, usual activities, and psychological status with three possible answers for each item (1=no problem, 2=moderate problem, 3=severe problem). A summary index with a maximum score of 1 can be derived from these five dimensions by conversion with a table of scores. The maximum score of 1 indicates the best health state, by contrast with the scores of individual questions, where higher scores indicate more severe or frequent problems. The visual analogue scale (VAS) indicates general health status with 100 indicating the best health status.

### Acceptability

The diagnostic assessments were acceptable to 395 (99%) of the cohort and all but two eligible participants consented to the trial; receipt of the HF intervention was endorsed by all consenting participants and care home staff as an experience they would be willing to repeat. Five GPs of residents receiving both intervention and control were interviewed as part of a qualitative evaluation (Close et al. 2012 in submission): all were supportive of the service.

## Discussion

This study challenges the status quo for HF management for older people in care. Findings of this pilot trial demonstrate the feasibility and applicability of evidence-based management in a group of people previously denied optimum care. International research over many decades has demonstrated the sub-optimal diagnosis and management of HF in older residents in care. Reasons for this include clinical reluctance to offer treatments that may have side-effects and which may ultimately reduce length and quality of life. In this study, the intervention group attained a higher level of evidence-based treatment of optimal doses of ACEi and β-blockers than the control group without deterioration in quality of life although results were non-significant due to the sample size. Around half of participants had a pre-existing diagnosis of LVSD and were receiving sub-optimal doses of ACEi and β-blockers but in this trial this was improved. It is possible that the magnitude of change over 6 months might have been even higher if treated cases of LVSD had been excluded in the trial, although drug therapy was already limited in this group.

The low numbers of hospitalisations in either group was surprising but reassuring in that the intervention group did not have increased admission rates. This counters often quoted fears of harm through higher (appropriate) doses and types of treatment. An important consideration for this trial was the length of time it would take to reach an appropriate level of treatment (in dose and type) for each patient. We found this could be achieved in the intervention group within 4 months, also suggesting the notional 6 month cut-off for assessment of the primary outcome was a reasonable comparison.

It remains uncertain whether treatment levels can be maintained in patients who had received the intervention following discharge back to usual care. Unless there is an ongoing and integrated approach to drugs management, improvements by one care provider may quickly be cancelled by changes made by another. For those discharged from the intervention, rates of prescribing of ACEi and β-blockers had dropped at 12 months following the trial. We were unable to ascertain reasons for the reduction. It would be helpful to understand why GPs altered personalised management plans in some patients when there were no indications that side effects or other drug-associated problems increased. In the qualitative arm of this trial (Close et al., in submission 2012), GPs expressed concern about comorbidities, polypharmacy and side-effects. Further understanding of these issues might allow us to draw definitive conclusions about models of care for heart failure management in the longer term. The introduction of a stand-alone intervention raises important questions about sustainability and capacity building in general practice. Additional steps might have been taken to improve GP knowledge about heart failure, however the purpose of this study was to assess baseline practices from which to inform the development of a subsequent structured interventional study. A subsequent (powered) study should evaluate new and current care pathways in primary care to inform effective heart failure diagnosis and management, measuring a range of end points (quality of life, mortality, and hospitalisation) as well as a cost-effectiveness analysis.

While the findings regarding short-term prescribing differences between GPs and the heart failure team might not be surprising, this is the first study to empirically demonstrate that additional support is required to raise standards of primary care for this group.

This trial comprised participants who had been screened for entry into the study and in whom HF status was ascertained. Rates of willingness to participate and retention during follow-up were very high but the trial may have recruited a selected population with less complex needs than the non-screened population. Whilst pilot findings should be interpreted with caution, baseline characteristics were collected for both screened and non-screened subjects. Similarities in these data suggest that selection bias was not a threat to this study.

This study excluded patients with HFpEF as it currently lacks a clearly evidence-based rationale for treatment [36]. Further work is needed to develop this evidence base from which to evaluate the potential for benefit in this group. Available research highlights the paucity of evidence for the effective therapeutic management of older people in care. While key trials [16,18,20,37] have assessed the efficacy of ACEi and β-blockers in LVSD and include older people, the mean age of participants is considerably lower than that of our cohort (84.2 years), thus the relevance of previous findings for this population is difficult to assess. Our experience exposes some of the challenges: recruitment to the trial was time consuming and resource intensive, most notably in accommodating the needs of physically and cognitively impaired older people. Barriers were apparent within each stakeholder group, with care home managers, clinicians, residents and carers challenging the appropriateness of research per se and of treatment for heart failure in particular. However, given the time and resources, it is possible and acceptable to involve older people in care homes in a detailed assessment process and trial. Further work is required to improve the accessibility of older people with cognitive difficulty and other comorbidities who wish to participate in research. The challenge now is for researchers and funders to recognise the extra resources, skills and time required to involve older people in research and evidence-based management, thus developing an appropriate evidence base for a group with neglected health needs.

## Conclusions

Our findings demonstrate the acceptability and feasibility of a tailored heart failure service for older people in care. This study also challenges an implicit orthodoxy that older people in care homes are unwilling or unable to engage in clinical research involving comprehensive diagnosis and management. Optimisation of medication appears possible and larger scale studies are required to quantify the scale of benefit.

## Competing interests

The authors declare that they have no competing interests.

## Authors’ contributions

HH: I declare that co-designed the study, led data collection, participated in data analysis, interpretation and writing this report. HC: I declare that I participated in data collection, data analysis, interpretation and writing this report. JMM: I declare that I co-devised the research question, co-designed the study, participated in data collection, led data analysis, interpretation and writing this report. JJM: I declare that I participated in data collection, data analysis, interpretation and writing this report. AF: I declare that I participated in data collection, data analysis, interpretation and writing this report. MdeB: I declare that I participated in data validation, interpretation and writing this report. TH: I declare that I participated in data collection. AB: I declare that I participated in data collection. DW: I declare that I verified data analyses. APSH: I declare that I co-devised the research question, co-designed the study, participated in data interpretation and writing this report. All authors have seen and approved the final manuscript.

## Pre-publication history

The pre-publication history for this paper can be accessed here:

http://www.biomedcentral.com/1471-2318/12/70/prepub
